# Circ_0058051 Targeted miR-129-5P Regulates Autophagy-Related Gene ATG7 to Promote the Inflammation of Gout

**DOI:** 10.1155/mi/6645479

**Published:** 2025-11-03

**Authors:** Jianwei Guo, Tianyi Lei, Yi Jiang, Peng Wang, Zeng Zhang, Xiang Yu, Guilin Jian, Quanbo Zhang, Yufeng Qing

**Affiliations:** ^1^Research Center of Hyperuricemia and Gout, Affiliated Hospital of North Sichuan Medical College, Nanchong 637000, China; ^2^Department of Geriatrics, Affiliated Hospital of North Sichuan Medical College, Nanchong 637000, China; ^3^Department of Emergency, Suining Central Hospital, Suining 629000, China; ^4^Department of Rheumatology and Immunology, Affiliated Hospital of North Sichuan Medical College, Nanchong 637000, China; ^5^Department of Emergency, The Third People's Hospital of Suining, Suining 629000, China

**Keywords:** ATG7, circ_0058051, gout, miR-129-5p, recurrence

## Abstract

Gout is a common autoinflammatory disease that clinically manifests as recurrent joint redness, swelling, and pain, but the molecular mechanism of recurrent gouty inflammation remains unclear. Circular RNAs (circRNAs) might exert their function by regulating autophagy. Our previous studies revealed that autophagy-related genes (ATGs) are differentially expressed in patients with acute gout. The aim of this study was to investigate the molecular mechanism by which circ_0058051 regulates autophagy as a competitive endogenous RNA (ceRNA) in recurrent gouty inflammation. Real-time quantitative PCR was used to measure the expression of circ_0058051, miR-129-5p, ATG7, LC3, and IL-1β. Western blotting was used to assess the protein levels of ATG7, LC3, and IL-1β. Enzyme-linked immunosorbent assay (ELISA) was used to measure the IL-1β, IL-6, and TNF-α levels. A dual-luciferase reporter assay was used to confirm the interaction between circ_0058051, miR-129-5p, and ATG7. The gout group expressed significantly more circ_0058051 and ATG7 and significantly less miR-129-5p than the HC group. In the 20 paired cases, compared with stable gout cases, the expression of circ_0058051 and ATG7 was significantly greater during a gout attack and even greater in patients with gout recurrence. The significant decrease in miR-129-5p expression was more pronounced in patients with gout recurrence. In the simulation model of gout recurrence in the peripheral blood of intercritical gout patients stimulated with MSU, circ_0058051 peaked 2 h after MSU stimulation, ATG7 peaked 1 h after MSU stimulation, and miR-129-5p expression was lowest 1 h after MSU stimulation. In addition, the expression levels of circ_0058051, ATG7, IL-1β, TNF-α, IL-6, and LC3 significantly increased after THP-1 macrophages were treated with MSU, and the expression of miR-129-5p significantly decreased. In MSU-stimulated THP-1 macrophages, circ_0058051 sponges miR-129-5p to promote the expression of the miR-129-5p target gene ATG7, leading to acute gout attack. Our findings suggest that circ_0058051 is involved in the recurrence of acute gout by targeting miR-129-5p to regulate ATG7-mediated autophagy.

## 1. Introduction

Gouty arthritis (GA) is a common autoimmune joint disease in which spontaneous relief and recurrent attacks are distinguishing features from other arthropathy or autoinflammatory diseases [1]. In recent years, many studies have focused on the mechanism of the spontaneous relief of gouty inflammation [2–5]; however, few reports have described the molecular mechanisms that trigger gouty inflammation recurrence. Autophagy, also known as Type II programmed cell death, is the process by which the body recycles material, maintains cellular metabolic balance and homeostasis [6, 7]. Moreover, autophagy is involved in the occurrence and development of inflammatory diseases, such as gout [8, 9]. However, the association between autophagy and the recurrence of gout is unclear.

Studies have suggested that noncoding RNAs (ncRNAs) are involved in the occurrence and development of diseases by regulating autophagy-related genes (ATGs) [10]. Circular RNAs (circRNAs) are single-stranded, covalently enclosed ncRNAs without a 5′ end cap or 3′ poly(A) tail structure that can act as molecular sponges for microRNAs (miRNAs) to mediate the expression of their downstream target genes [11]. circRNAs participate in the pathological processes of cancers [12], cardiovascular diseases [13], and inflammatory diseases [14]. Our previous microarray studies identified aberrant circRNA profiles in gout patients, including high circ_0058051 expression [15]. circ_0058051 is produced from exons of the BARD1 gene on chromosome 2 and has a length of 1410 bp [16]. Bioinformatics analysis revealed that circ_0058051 acts as a competitive endogenous RNA (ceRNA) of miR-129-5p to competitively inhibit ATG7. Previous studies by our group revealed abnormal circ_0058051, miR-129-5p, and ATG7 expression in patients with gout. Is circ_0058051 involved in gout inflammation recurrence via a ceRNA mechanism?

**Figure 1:**
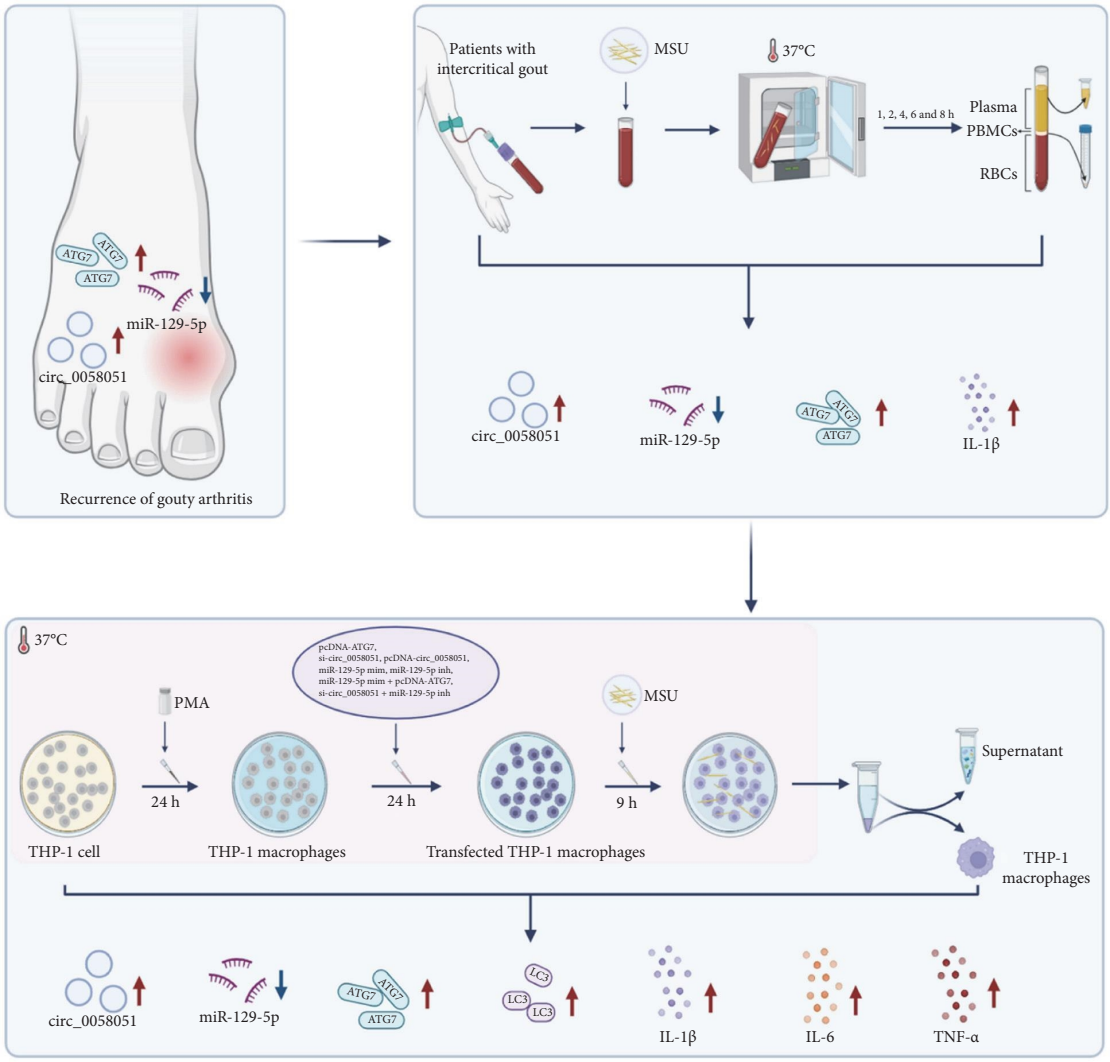
The experimental procedure of our study.

In this study, we examined the expression of circ_0058051, miR-129-5p, and ATG7 in peripheral blood mononuclear cells from gout patients at three different stages of inflammation, that is, the first attack, stabilization, and inflammatory recurrence. We found that among the three, circ_0058051 and ATG7 expression were highest and miR-129-5p expression was lowest in the PBMCs of patients with gout-related inflammation recurrence. We hypothesized that circ_0058051, miR-129-5p, and ATG7 are involved in the molecular mechanism of gout inflammation recurrence. Next, we explored the function of the circ_0058051/miR-129-5p/ATG7 axis by simulating inflammatory gout recurrence in vitro. To this end, we treated the peripheral blood of patients with intercritical gout with MSU crystals and either overexpressed or knocked down circ_0058051, miR-129-5p, and ATG7 in THP-1 macrophages. We found that the circ_0058051/miR-129-5p/ATG7 axis may be involved in the molecular mechanism of inflammatory gout recurrence. The experimental procedure of our study is shown in Figure 1.

## 2. Materials and Methods

### 2.1. Clinical Samples

Fifty male gout patients who were treated at the Department of Rheumatology and Immunology of the Affiliated Hospital of North Sichuan Medical College from November 2022 to April 2023 were recruited. All patients met the 2015 American College of Rheumatology/European League Against Rheumatism (ACR/EULAR) Classification Criteria for gout. Another 20 gout patients were selected, and their clinical data and peripheral venous blood were collected at the time of the first attack, stabilization, and recurrence. Specifically, patients with symptoms such as joint pain, swelling, and fever within 3 days after the first gout attack were included in the first attack group; those who were free of joint symptoms for at least 2 weeks after treatment and had high sensitivity C-reactive protein levels, erythrocyte sedimentation rates, and white blood cell counts within normal ranges were included in the stabilization group. Patients with gout in whom arthritis symptoms recurred and whose clinical indicators were elevated were included in the recurrence group. During the same period, another 50 healthy (HC) men without metabolic syndrome, hyperuricemia, or other chronic diseases were recruited from the Physical Examination Centre of the Affiliated Hospital of North Sichuan Medical College. Laboratory parameters were recorded for all the subjects (Tables S1 and S2), and single nucleated cells were isolated from the peripheral blood of gout patients and healthy controls via Ficoll‒Paque PLUS (GE Healthcare, USA) and stored at ‒80°C. All participants provided informed consent. This study was approved by the Ethics Committee of the Affiliated Hospital of North Sichuan Medical College (Number 2022ER463-1).

### 2.2. MSU Preparation

MSU was prepared as described in a previous report. Uric acid (1 g; Sigma, USA) was dissolved in 200 mL of boiling water containing 6 mL of 1 N NaOH. The pH value of the final solution was adjusted to 7.2 by adding hydrochloric acid. The solution was cooled, stirred at room temperature, and stored overnight at 4°C. The sediment was filtered out of the solution and dried at low heat. The crystals were weighed and suspended in PBS under sterile conditions to a concentration of 25 mg/mL [17].

### 2.3. MSU Stimulation of Peripheral Venous Blood in Patients With Intercritical Gout

Peripheral venous blood (24 mL, 4 mL/tube) was collected from five male patients with intercritical gout, anticoagulated with heparin sodium, stimulated with 100 µg/mL MSU, and incubated in 5% CO_2_ at 37°C. The plasma was isolated, and the PBMCs were extracted 0, 1, 2, 4, 6, and 8 h after stimulation.

### 2.4. Cell Culture

Human myeloid leukemia mononuclear cells (THP-1) were purchased from Procell Life Science and Technology (Procell, China) and incubated in RPMI 1640 medium containing 10% foetal bovine serum (Thermo Fisher Scientific, USA) and 1% penicillin‒streptomycin (Thermo Fisher Scientific, USA) at 37°C in a 5% CO_2_ humidified incubator. THP-1 cells were differentiated with 100 ng/mL phorbol 12-myristate 13-acetate/ionomycin (PMA; Sigma, USA) for 48 h to induce monocyte-to-macrophage conversion and were then stimulated with 100 µg/mL MSU for 3, 6, 9, and 12 h. The supernatant and cells were collected.

### 2.5. Cell Transfection

To knock out circ_0058051, small interfering RNAs that target circ_0058051 (si-circ_0058051), including si-circ_0058051#1, si-circ_0058051#2, and si-circ_0058051#3, and a negative control (si-NC), were synthesized by RiboBio (Guangzhou, China). To overexpress circ_0058051, a circ_0058051-overexpression vector and a negative control (pcDNA) were synthesized by Jikai (Shanghai, China). Mimics or inhibitors that target miR-129-5p (miR-129-5p mim or miR-129-5p inh) and the corresponding negative controls (NC-mim and NC-inh) were purchased from RiboBio (Guangzhou, China). To overexpress ATG7, the ATG7 sequence was cloned and inserted into the pcDNA vector (ATG7); both the overexpression plasmid and the control (vector) were synthesized by RiboBio (Guangzhou, China). The cells were transfected with Lipofectamine 2000 (Invitrogen, USA).

### 2.6. RNA Extraction and Real-Time Quantitative Polymerase Chain Reaction (RT-qPCR)

Total RNA was extracted via the TRIzol method. Subsequently, circ_0058051 and ATG7 cDNA were synthesized via the PrimeScript RT Kit (Takara, China). The primer sequences are shown in Table S3. miR-129-5p cDNA was synthesized with the One-Step PrimeScript-miRNA cDNA Kit (Takara, China). RT-qPCR was performed using SYBR Premix Ex Taq II (Takara, China). The relative expression of the target gene was calculated via the 2^−*ΔΔ*CT^ method. β-Actin and U6 were used as standardized internal controls.

### 2.7. Western Blotting

Total protein was extracted with RIPA buffer and then quantified with a BCA protein assay kit (Biyuntian, China). The extracted proteins were separated by 10% SDS-PAGE and then transferred to polyvinylidene difluoride membranes (Millipore, USA). Fast blocking solution (Biyun Tian, China) was used for 20 min, after which the membranes were incubated with primary antibodies against ATG7 (ab133528, 1:10000, Abcam, China), LC3 (#3868, 1:1000, Cell Signaling Technology, USA), IL-1β (#63124, 1:1000, Cell Signaling Technology, USA), and GAPDH (#5174, 1:1000, Cell Signaling Technology, USA) overnight at 4°C. The membranes were then incubated with a horseradish peroxidase-conjugated secondary antibody (1:1000, Abcam, China) at room temperature for 1 h. Finally, ECL reagent (Affinity, USA) was used to visualize the protein bands on a gel imaging system.

### 2.8. Dual-Luciferase Reporter Assay

Circ_0058051, ATG7 wild-type luciferase reporting vector (circ_0058051 WT, ATG7 WT) and circ_0058051, ATG7 mutated luciferase reporting vector (circ_0058051 mut; Boyuan, China) were constructed. Then, the above vectors were cotransfected with the miR-129-5p mimic and NC-mim into HEK293T cells using Lipofectamine 2000. Forty-eight hours after transfection, luciferase activity was analyzed with a dual luciferase assay kit (Promega, Shanghai, China).

### 2.9. Enzyme-Linked Immunosorbent Assay (ELISA)

The supernatant was collected from the peripheral blood of patients in the intercritical period of gout after stimulation by MSU, as well as from the supernatant of MSU-stimulated THP-I macrophages. Secreted cytokines, such as IL-1β, TNF-α, and IL-6, were analyzed with ELISA kits (Wuhan Yunkun Company, China).

### 2.10. Statistical Analysis

Statistical analysis was performed with SPSS 23.0. Quantitative data with approximate normal distribution was expressed as mean ± standard deviation and nonnormal distribution were expressed as median. Intergroup comparisons were conducted using analysis of variance (ANOVA), and between two groups via the independent sample *t* test. *p*  < 0.05 was considered a statistically significant difference.

## 3. Results

### 3.1. Altered Expression of circ_005801, miR-129-5p, and ATG7 in Different Inflammatory Stages of Gout

**Figure 2:**
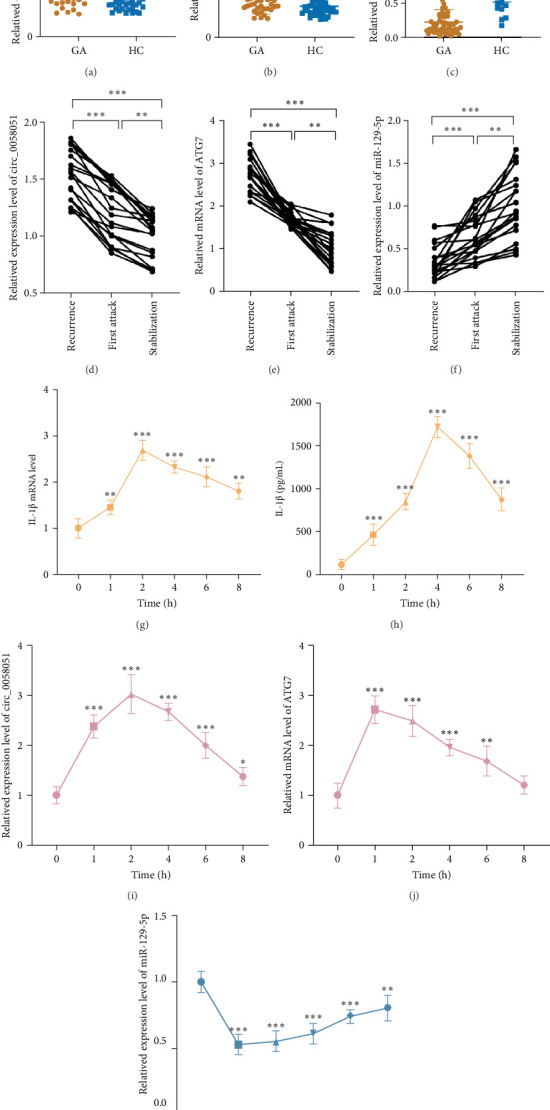
Altered expression of circ_005801, miR-129-5p, and ATG7 in different inflammatory stages of gout patients. (a–c) Expression of circ_0058051, miR-129-5p, and ATG7 in PBMCs of gout patients. (d–f) Expression of circ_0058051, miR-129-5p, and ATG7 at different periods of gouty attacks in 20 cases using RT-qPCR. (g) Expression of IL-1β in peripheral blood of intercritical gout patients with MSU-stimulated after 0, 1, 2, 4, 6, and 8 h were detected by RT-qPCR. (h) Expression of IL-1β in peripheral blood of intercritical gout patients with MSU-stimulated after 0, 1, 2, 4, 6, and 8 h were detected by ELISA. (i–k) Expression of IL-1β, circ_0058051, miR-129-5p, and ATG7 in peripheral blood of intercritical gout patients with MSU-stimulated after 0, 1, 2, 4, 6, and 8 h were detected by RT-qPCR. *⁣*^*∗*^*p* < 0.05, *⁣*^*∗∗*^*p* < 0.01, *⁣*^*∗∗∗*^*p* < 0.001.

The expression levels of circ_0058051 and ATG7 were significantly greater in the GA group than in the HC group, and the expression of miR-129-5p was significantly lower in the gout group than in the HC group (Figure 2a–c). Notably, the expression levels of circ_0058051 and ATG7 were significantly greater and the expression level of miR-129-5p was significantly lower in patients with recurrent gout than in the 20 patients with a first gout attack (Figure 2d–f). The peripheral blood of intercritical gout patients was stimulated with MSU to mimic the recurrence of acute GA in vitro. Supernatants and cells were collected at 0, 1, 2, 4, 6, and 8 h. Compared with those at 0 h (no stimulation), the expression levels of IL-1β, circ_0058051, and ATG7 were significantly greater after MSU stimulation and peaked in the early stage of MSU stimulation, whereas the expression level of miR-129-5p was lower and reached a minimum 1 h of MSU stimulation (Figure 2g–k). These results suggest that MSU induces inflammation in intercritical gout patients and that circ_0058051, miR-129-5p, and ATG7 might regulate the recurrence of GA.

### 3.2. Altered Expression of circ_0058051, miR-129-5p, ATG7, and Cytokines in MSU-Stimulated THP-1 Macrophages

**Figure 3:**
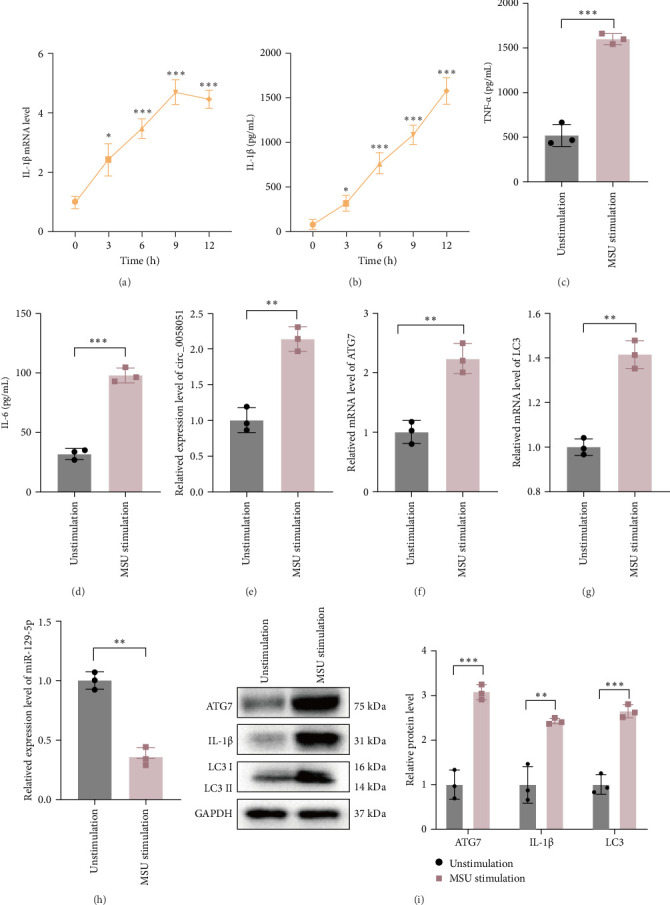
Altered expression of circ_0058051, miR-129-5p, ATG7, and cytokines in MSU-induced THP-1 macrophages. (a) Expression of IL-1β in MSU-stimulated THP1 cells after 0, 3, 6, 9, and 12 h was detected by RT-qPCR. (b) The protein concentration of IL-1β in MSU-stimulated THP1 cells after 0, 3, 6, 9, and 12 h was measured by ELISA assay. (c, d) The protein concentrations of TNF-α and IL-6 in THP-1 macrophages stimulated by MSU were detected by ELISA. (e–h) The expressions of posterior circ_0058051, miR-129-5p, ATG7, and LC3 in MSU-stimulated THP-1 macrophages were detected by RT-qPCR. (i) The protein expressions of ATG7, IL-1β, and LC3 in THP-1 macrophages were detected by western blot 9 h after MSU stimulation of THP-1 macrophages. The data are expressed as means ± SEM of at least three independent experiments. *⁣*^*∗*^*p* < 0.05, *⁣*^*∗∗*^*p* < 0.01, *⁣*^*∗∗∗*^*p* < 0.001.

A model of acute GA was constructed in vitro by stimulating THP-1 macrophages with MSU crystals. The RT-qPCR results revealed that IL-1β mRNA expression peaked at 9 h of MSU stimulation and that the IL-1β protein expression level continued to increase after 9 h (Figure 3a,b), suggesting that inflammation in acute GA may peak at 9 h. Therefore, 9 h was selected as the time point for subsequent experiments. Compared with those in the blank control group, the protein concentrations of TNF-α and IL-6 were significantly greater in the supernatants of cultures of THP-1 macrophages stimulated with MSU (Figure 3c,d). The expression levels of circ_0058051, ATG7, and LC3 significantly increased after MSU stimulation, and the expression of miR-129-5p significantly decreased (Figure 3e‒i). These results indicate that the abnormal expression of circ_0058051, miR-129-5p, and ATG7 may be related to the inflammatory response in gout.

### 3.3. miR-129-5p Links ATG7 and circ_0058051

**Figure 4:**
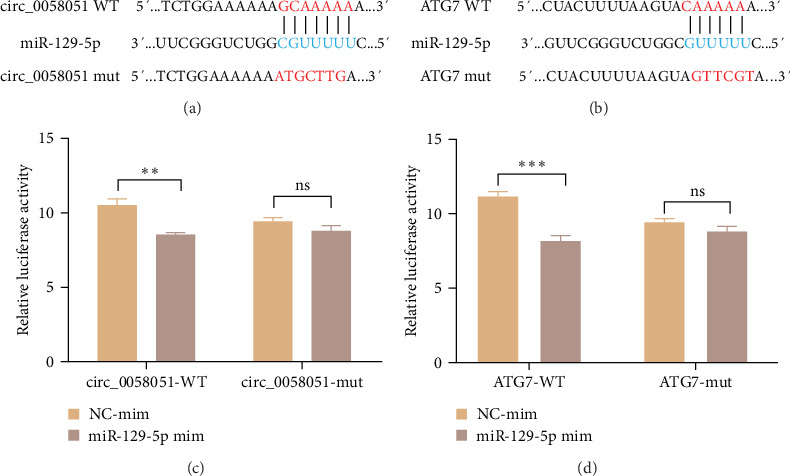
miR-129-5p links ATG7 and circ_0058051. (a) Predicted binding site of circ_0058051 to miR-129-5p. (b) Predicted binding site of miR-129-5p to ATG7. (c) Dual luciferase reporter gene analysis of the interaction between circ_0058051 and miR-129-5p. (d) Dual luciferase reporter gene analysis of the interaction between miR-129-5p and ATG7. The data are expressed as means ± SEM of at least three independent experiments. *⁣*^*∗∗*^*p* < 0.0, *⁣*^*∗∗∗*^*p* < 0.001.

Studies have reported that circRNAs can be involved in regulating the occurrence and progression of diseases as ceRNAs [18], but whether circ_0058051 acts as a ceRNA in gout is currently unknown. In this study, bioinformatics tools (CircInteractome, miRDB, and TargetScan) were used to predict the interaction relationships, and a binding site was identified between circ_0058051 and miR-129-5p (Figure 4a). In addition, a potential binding site in ATG7 for miR-129-5p was also identified (Figure 4b). The results of a dual-luciferase reporter gene assay revealed that miR-129-5p significantly reduced the luciferase activity of wild-type circ_0058051 and wild-type ATG7 but that the luciferase activities of mutant circ_0058051 and mutant ATG7 were not affected by miR-129-5p (Figure 4c,d). These results indicate that miR-129-5p is a circ_0058051 target and that ATG7 is a miR-129-5p target.

### 3.4. Effect of ATG7 on Cytokine Secretion in MSU-Stimulated THP-1 Macrophages

**Figure 5:**
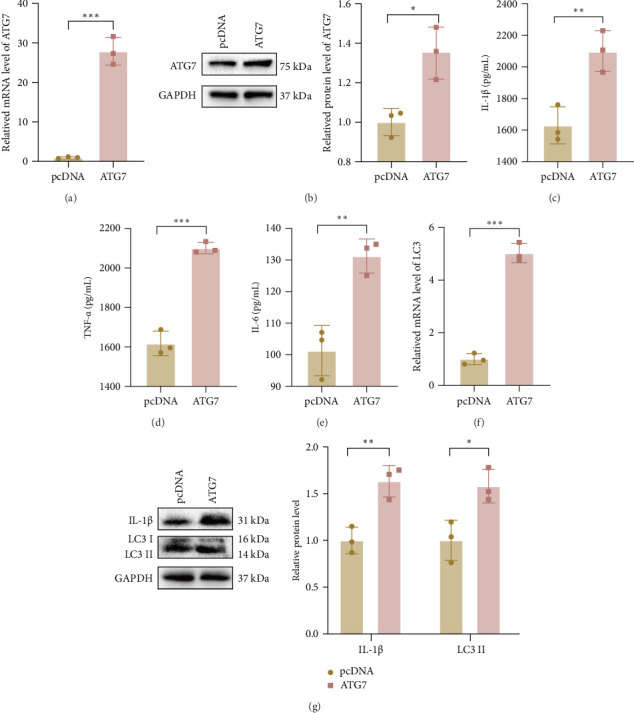
Effect of ATG7 on cytokine secretion in MSU-stimulated THP-1 macrophages. (a, b) The efficiency of transfection of THP-1 macrophages with ATG7 overexpression plasmid was verified by RT-qPCR and western blot. (c, d) MSU-stimulates ATG7 overexpression plasmid to transfect THP-1 macrophages. (c–e) The protein concentrations of IL-1β, TNF-a, and IL-6 were detected by ELISA. (f) The expression of LC3 was detected by RT-qPCR. (g) The protein expression levels of IL-1β and LC3 were detected by Western blot. The data are expressed as means ± SEM of at least three independent experiments. *⁣*^*∗*^*p* < 0.05, *⁣*^*∗∗*^*p* < 0.01, *⁣*^*∗∗∗*^*p* < 0.001.

To clarify the role of ATG7 in the progression of gout, we transfected THP-1 macrophages with an ATG7 overexpression plasmid and verified the transfection efficiency (Figure 5a,b). The protein concentrations of IL-1β, TNF-α, and IL-6 were elevated in the supernatants of the ATG7-overexpressing THP-1 macrophages, and the expression level of LC3 was significantly elevated (Figure 5c–g). These results suggest that ATG7 positively regulates the production of inflammatory cytokines and promotes the inflammatory response during MSU-induced inflammation.

### 3.5. Effects of the miR-129-5p/ATG7 Axis on Autophagy and Cytokine Secretion in MSU-Stimulated THP-1 Macrophages

**Figure 6:**
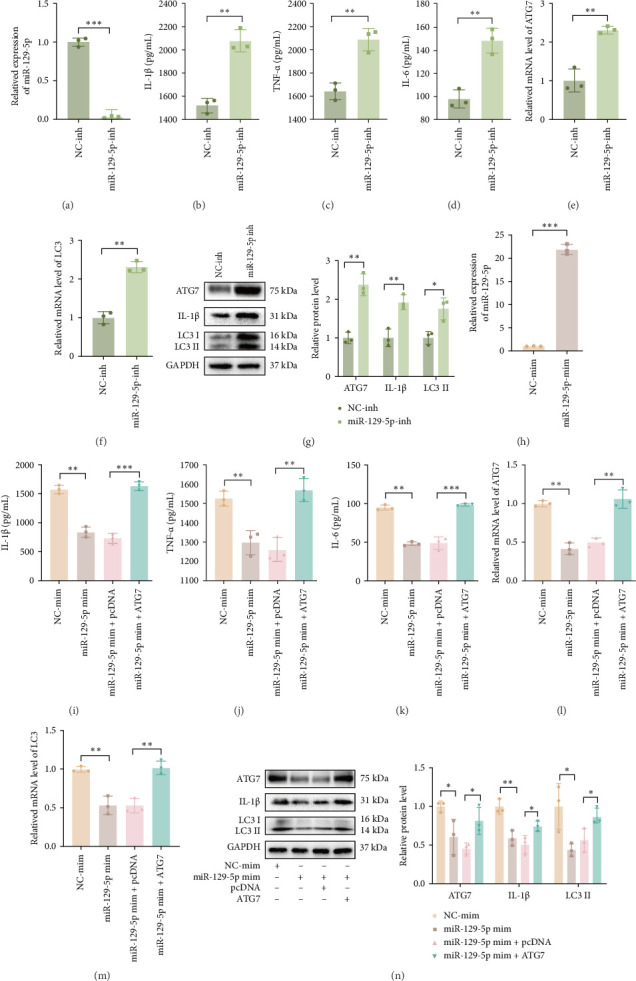
Effect of the miR-129-5p/ATG7 axis on autophagy and cytokine secretion in MSU-stimulated THP-1 macrophages. (a) The transfection efficiency of THP-1 macrophages treated with miR-129-5p inh was verified by RT-qPCR. (b–h) miR-129-5p inh-treated THP-1 macrophages were stimulated using MSU. (b–d) Protein concentrations of IL-1β, TNF-a, and IL-6 were detected using ELISA. (e, f) The expression of ATG7 and LC3 were detected by RT-qPCR. (g) The protein expression levels of ATG7, IL-1β, and LC3 were detected by western blot. (h) The transfection efficiency of THP-1 macrophages treated with miR-129-5p mim was verified using RT-qPCR. (i–n) THP-1 macrophages transfected with NC-mim, miR-129-5p mim, miR-129-5p mim + pcDNA, and miR-129-5p mim + ATG7 were stimulated with MSU;. (i–k) Protein concentrations of IL-1β, TNF-a, and IL-6 were detected using ELISA. (l, m) The expression of ATG7 and LC3 was detected by RT-qPCR. (n) The protein expression levels of ATG7, IL-1β, and LC3 were detected by western blot. The data are expressed as means ± SEM of at least three independent experiments. *⁣*^*∗*^*p* < 0.05, *⁣*^*∗∗*^*p* < 0.01, *⁣*^*∗∗∗*^*p* < 0.001.

To investigate whether ATG7 is regulated by miR-129-5p in MSU-stimulated inflammation, THP-1 macrophages were transfected with miR-129-5p inh, and as expected, miR-129-5p inh decreased miR-129-5p expression in THP-1 macrophages (Figure 6a). The expression levels of IL-1β, TNF-α, IL-6, ATG7, and LC3 II significantly increased in MSU-stimulated THP-1 macrophages transfected with miR-129-5p inh (Figure 6b–g). Subsequently, the miR-129-5p mimics were transfected into THP-1 macrophages and the transfection efficiency was verified (Figure 6h). The expression levels of IL-1β, TNF-α, IL-6, ATG7, and LC3 II significantly decreased in THP-1 macrophages transfected with miR-129-5p mimics. In addition, we also found that the decreases in IL-1β, TNF-α, IL-6, ATG7, and LC3 levels caused by the upregulation of miR-129-5p were reversed by the increase in ATG7 (Figure 6i–n). These results indicate that ATG7 promotes autophagy and that the inflammatory response in THP-1 macrophages is inhibited by miR-129-5p.

### 3.6. The miR-129-5p/ATG7 Axis Affects the Inflammatory Response and Autophagy in MSU-Stimulated THP-1 Macrophages Regulated by circ_0058051

**Figure 7:**
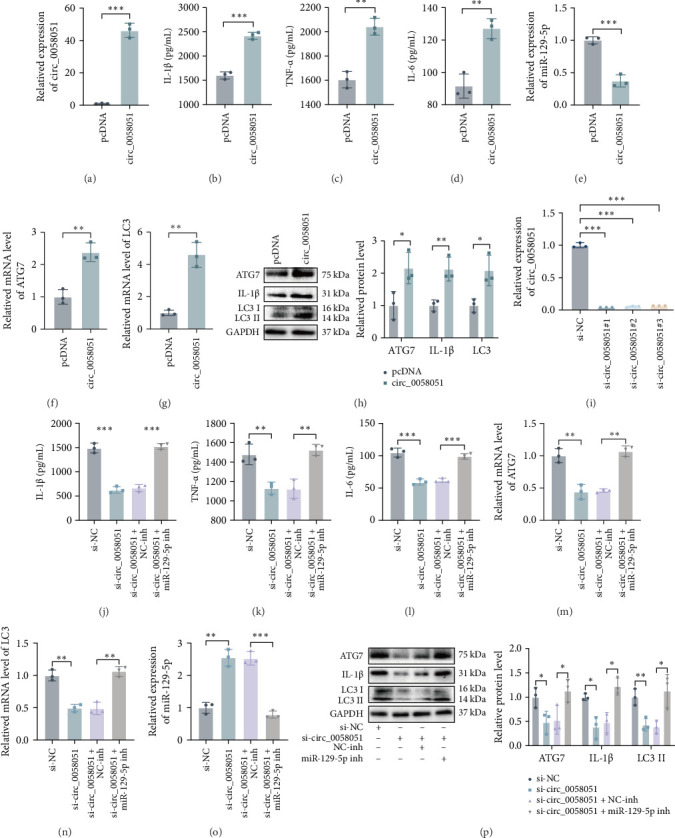
The miR-129-5p/ATG7 axis affects the inflammatory response and cellular autophagy in MSU-stimulated THP-1 macrophages regulated by circ_0058051. (a) Transfection efficiency of THP-1 macrophages circ_0058051 overexpression plasmid was verified using RT-qPCR. (b–h) MSU stimulates THP-1 macrophages transfected circ_0058051 overexpression plasmid. (b–d) Protein concentrations of IL-1β, TNF-a, and IL-6 were detected using ELISA. (e–g) The expression of miR-129-5p, ATG7, and LC3 were detected by RT-qPCR. (h) The protein expression levels of ATG7, IL-1β, and LC3 were detected by western blot. (i) The efficiency of si-circ_0058051 transfection of THP-1 macrophages was verified using RT-qPCR. (j–p) THP-1 macrophages transfected with si-NC, si-circ_0058051, si-circ_0058051+NC-inh, and si-circ_0058051 + miR-129-5p inh were stimulated with MSU. (j–l) Protein concentrations of IL-1β, TNF-a, and IL-6 were detected using ELISA. (m–o) The expression of ATG7 and LC3 were detected by RT-qPCR. (p) The protein expression levels of ATG7, IL-1β, and LC3 were detected by western blot. The data are expressed as means ± SEM of at least three independent experiments. *⁣*^*∗*^*p* < 0.05, *⁣*^*∗∗*^*p* < 0.01, *⁣*^*∗∗∗*^*p* < 0.001.

We performed gain-of-function experiments using circ_0058051-overexpressing plasmids to transfect THP-1 macrophages, and the transfection efficiency was verified (Figure 7a). The protein concentrations of IL-1β, TNF-α, and IL-6 were significantly increased, the expression levels of ATG7 and LC3 were increased, and the expression levels of miR-129-5p were decreased in circ_0058051-overexpressing THP-1 macrophages stimulated with MSU (Figure 7b–h). We further performed functional loss experiments by transfecting si-circ_0058051#1, si-circ_0058051#2, and si-circ_0058051#3 into THP-1 macrophages. The transfection efficiency was verified, and si-circ_0058051#1 was selected for transfection in subsequent experiments because it exhibited the highest transfection efficiency (Figure 7i). In THP-1 macrophages transfected with si-circ_0058051 and stimulated with MSU, the expression levels of IL-1β, TNF-α, IL-6, ATG7, and LC3 were decreased, and the expression levels of miR-129-5p were significantly increased. In addition, we also found that the circ_0058051-mediated decreases in IL-1β, IL-6, TNF-α, and LC3 were reversed by a decrease in miR-129-5p (Figure 7j–p). These results indicate that circ_0058051 regulates ATG7 expression in MSU-stimulated THP-1 macrophages by sponging miR-129-5p, thereby promoting cellular autophagy and inflammatory responses.

## 4. Discussion

MSU crystals are the key material basis for the occurrence of gouty inflammation [19, 20]. Macrophages engulf MSU crystals to activate inflammatory signaling pathways mediated by innate immune toll-like receptors and NOD-like receptors. Inflammatory factors (such as IL-1β and TNF-α) and proteases are released, which interact with peripheral nociceptors to cause severe pain [21–23]. Recurrent attacks and spontaneous relief are the distinguishing features of gout [24]. Recent studies have shown that the self-limiting nature of gouty inflammation may be related to neutrophil extracellular traps, apoptosis, diet, and other factors [3, 25]. However, the exact molecular mechanism of gout recurrence remains unclear and is one of the problems to be solved with respect to the pathogenesis of gout. Recent studies have shown that autophagy and circRNAs play crucial roles in the occurrence and development of gout [26–28]. This study is the first to confirm that circ-0058051 promotes ATG7 expression by inhibiting miR-129-5p, which promotes gout recurrence. Autophagy is a protein degradation mechanism in eukaryotic cells, and LC3 is considered a specific marker protein for autophagosome formation [28]. MSU crystals promote the formation of autophagosomes and induce the degradation of damaged proteasomes, thereby increasing the levels of the inflammatory factor IL-1β [29]. Through in vitro cell experiments, Mitroulis et al. [30] reported that MSU stimulated the activation of the NLRP3 inflammasome, which was accompanied by an increase in autophagy activity. ATG7 couples to PE on the surface of the autophagosome membrane to form a membrane-bound form of LC3 II and drive autophagosomal membrane expansion and vesicle formation [31]. The role of ATG7 in IL-1β release is downstream of NLRP3 inflammasome assembly and caspase-1 cleavage of pro-IL-1β [32]. In this study, we found that ATG7 levels were significantly increased in gout patients. Furthermore, the expression of ATG7 was significantly increased during the recurrence period in in PMBCs collected from gout patients compared to PMBCs collected after the first attack or the stabilization of treatment. To reduce the error caused by different sample collection times, we used MSU to stimulate peripheral blood from patients with intercritical gout to simulate the recurrence of acute GA in vitro. We found that the expression level of ATG7 increased sharply in the early stage after MSU stimulation. Interestingly, the level of IL-1β also peaked 2 h after MSU stimulation. ATG7 may induce excessive autophagy and activate NLRP3 to release inflammatory cytokines during gout recurrence. According to a previous report, macrophages mediate the entire process of acute gout, and monocyte-derived macrophages differentiate into M1-like macrophages in the presence of MSU crystals [33]. Therefore, we used PMA to induce THP-1 cells to differentiate into macrophages and then stimulated the THP-1 macrophages with MSU to establish an in vitro model of acute GA. The protein concentrations of inflammatory cytokines (IL-1β, TNF-α, and IL-6) significantly increased after ATG7 overexpression in MSU-stimulated THP-1 macrophages, suggesting that ATG7 may be involved in the mechanism of gout recurrence by promoting the release of inflammatory cytokines.

In recent years, miRNAs have been shown to regulate cell autophagy [34]. Mature miRNAs can bind to complementary sequences in the 3′ untranslated region of mRNAs, thereby inhibiting translation or promoting mRNA degradation [35]. We found that ATG7 is a target of miR-129-5p. miR-129-5p has been reported to be involved in regulating the release of inflammatory factors, the overexpression of which prevents excessive activation of inflammation in acute kidney injury [36]. Qiu et al. [37] reported that a reduction in miR-129-5p in exosomes secreted by mesenchymal stem cells increased IL-1β-mediated inflammation and apoptosis in chondrocytes. In heart failure, overexpression of ATG7 reversed the cardiomyocyte apoptosis and autophagy inhibition mediated by miR-129-5p mimics [38]. miR-129-5p may act on the autophagy signaling network related to ATG7, thereby regulating the generation of white and brown adipocytes [39]. In this study, miR-129-5p expression was reduced in patients with gout recurrence and in an in vitro model of acute GA. It is involved in regulating autophagy and the inflammatory response of THP-1 macrophages, suggesting that miR-129-5p may be involved in the occurrence and development of gout recurrence through negative regulation. In addition, we observed that ATG7 was negatively regulated by miR-129-5p, which inhibited the inflammatory response and autophagy in gout by inhibiting the expression of ATG7. The results of our study suggest that ATG7 is a key downstream factor in the inflammatory response inhibited by miR-129-5p in gout recurrence.

circRNAs are inherently conserved, highly stable, and can act as molecular sponges that bind to miRNAs and isolate and competitively inhibit miRNA activity [40]. Dai et al. [26] analyzed transcriptome sequencing data from gout patients via genome-wide microarrays and reported that circRNAs may be involved in the pathogenesis of gout through inflammatory and immune pathways and that hsa_circRNA_103657 and hsa_circRNA_000241 may affect gout inflammation through multiple pathways. A study by Lian et al. [41] revealed that circHIPK3 promotes the expression of TLR4 and NLRP3 by sponging miR-192 and miR-561, thereby promoting inflammatory signaling pathways and increasing inflammatory cytokine levels in GA. Dual-luciferase reporter gene analyses revealed that circ_0058051 and miR-129-5p have a targeted binding relationship. circ_0058051 has been confirmed to be highly expressed in the peripheral blood of gout patients [15]. In this study, circ_0058051 was more highly expressed in the peripheral blood of patients with recurrent gout and in the MSU-stimulated intermittent phase of gout. In addition, circ_0058051 was found to promote autophagy and the inflammatory response in MSU-stimulated THP-1 macrophages, and the upregulation of circ_0058051 was hypothesized to increase the release of inflammatory cytokines and induce autophagy. Silencing circ_0058051 inhibited the expression of ATG7 through miR-129-5p, thereby inhibiting autophagy and inflammatory responses in MSU-stimulated THP-1 macrophages. These findings suggest that circ_0058051 may regulate ATG7-mediated autophagy by sponging miR-129-5p, thereby promoting inflammatory responses during gout recurrence.

**Figure 8:**
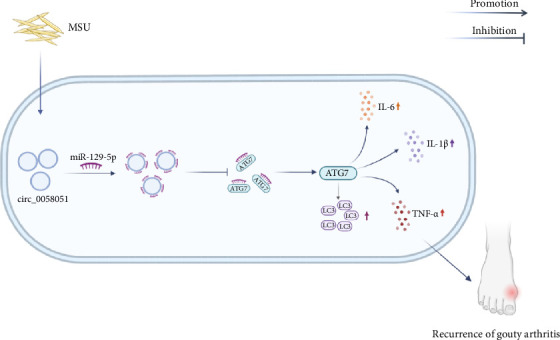
circ_0058051 may be involved in the recurrence of gout inflammation by targeting miR-129-5p to regulate ATG7-mediated autophagy.

We report, for the first time, that circ_0058051 induces recurrent episodes of GA by targeting miR-129-5p to regulate ATG7-mediated autophagy to promote the production of inflammatory cytokines. As depicted in the mechanistic diagram in Figure 8, circ_0058051 binds to miR-129-5p to inhibit its binding to ATG7, resulting in an increase in ATG7, thereby increasing the ATG7-mediated release of autophagy and inflammatory cytokines. These results suggest that targeting the circ_0058051/miR-129-5p/ATG7 axis may be an effective therapeutic strategy to prevent the recurrence of gout. Notably, this study has several limitations. First, the sample size was relatively small. At present, the findings of this study have been validated at the cellular level, but the regulatory mechanism in vivo is far more complex than that in vitro. In the future, we will further validate our findings by constructing an animal model for in vivo experiments.

## 5. Conclusion

Our study suggests that the circ_0058051/miR-129-5p/ATG7 axis may promote the recurrence of acute GA and that circ_0058051 may act as a sponge for miR-129-5p to regulate ATG7-mediated autophagy, which is important in the reoccurrence of GA. The circ_0058051/miR-129-5p/ATG7 axis is expected to be a new target for the prevention of gout recurrence.

## Data Availability

The original contributions presented in the study are included in the article, further inquiries can be directed to the corresponding author.

## Nomenclature

GA: Gouty arthritis
HC: Healthy group
miR-129-5p inh: miR-129-5p inhibitors
miR-129-5p mim: miR-129-5p mimics
ATG7: Autophagy related protein 7
LC3: Microtubule-associated protein 1 light chain 3
IL-1β: Interleukin-1 beta
IL-6: Interleukin-6
TNF-α: Tumor necrosis factor-α
ncRNAs: Noncoding RNAs
circRNAs: Circular RNAs
miRNAs: MicroRNAs
RT‒qPCR: Real-time quantitative polymerase chain reaction
ELISA: Enzyme-linked immunosorbent assay.

## Supporting Information

Supporting Information Table S1: Laboratory indexes of subjects in GA group and HC group. Table S2: Indicators of laboratory examination in 20 matched gout patients. Table S3: Real-time fluorescent quantitative PCR primers.
