# Supplementary material for: Circ_0058051 Targeted miR-129-5P Regulates Autophagy-Related Gene ATG7 to Promote the Inflammation of Gout
Source: Mediators Inflamm. 2025 Nov 3;2025:6645479. doi: 10.1155/mi/6645479 (PMC12602026; doi:10.1155/mi/6645479)
Supplement: Supporting Information — Table S1: Laboratory indexes of subjects in GA group and HC group. Table S2: Indicators of laboratory examination in 20 matched gout patients. Table S3: Real-time fluorescent quantitative PCR primers. [file 6645479.f1.docx]

**Supplementary Material**

**Table S1 Laboratory indexes of subjects in GA group and HC group**

| **Laboratory Indexes** | **GA(n=50)** | **HC (n=50)** |
| --- | --- | --- |
| **ESR(mm/1 h)** | 30.08±19.02 | - |
| **CRP(mg/L)** | 15.58±13.70 | - |
| **WBC(×10^9^/L)** | 8.04±2.60^a^ | 5.95±1.28 |
| **GR(×10^9^/L)** | 5.51±2.35^a^ | 3.51±0.76 |
| **LY(×10^9^/L)** | 2.20±0.63 | 2.07±0.43 |
| **MO(×10^9^/L)** | 0.49±0.19^a^ | 0.37±0.09 |
| **Crea(mol/L)** | 87.28±21.47^a^ | 66.98±5.86 |
| **sUA(umol/l)** | 484.48±112.43^a^ | 331.39±24.15 |
| **TG(mmol/L）** | 1.34±0.48 | 1.32±0.54 |
| **TC(mmol/L）** | 4.87±0.86 | 4.80±0.68 |
| **Glu（mmol/L）** | 5.19±0.58 | 4.94±0.82 |

ESR, rythrocyte sedimentation rates; CRP, c-reactive protein; WBC, white blood cell counts; GR, Neutrophil counts; LY, Lymphocyte; MO, Monocyte; Crea, serum creatinine; sUA, serum uric acid; TG, triglycerides; TC, total cholesterol; Glu, blood glucose; ^a^ *P* < 0.05 (in comparison with the HC group).

**Table S2 Indicators of laboratory examination in 20 matched gout patients**

| **Laboratory Indexes** | **Recurrence**  **(n=20)** | **First attack**  **(n=20)** | **Stabilization**  **(n=20)** |
| --- | --- | --- | --- |
| **ESR(mm/1 h)** | 33.85±11.92^b^ | 29.75±13.45^b^ | 12.05±6.46 |
| **CRP(mg/L)** | 15.40±8.58^b^ | 15.62±9.64^b^ | 3.14±1.86 |
| **WBC(×10^9^/L)** | 9.83±1.80^b^ | 9.47±1.95^b^ | 6.91±1.47 |
| **GR(×10^9^/L)** | 5.37±1.26 | 5.40±1.31 | 5.09±1.18 |
| **LY(×10^9^/L)** | 2.40±0.92 | 2.34±1.11 | 2.34±1.09 |
| **MO(×10^9^/L)** | 0.48±0.12 | 0.49±0.10 | 0.46±0.11 |
| **Crea(mol/L)** | 84.43±25.93 | 83.37±28.21 | 83.72±25.23 |
| **UA(umol/l)** | 519.60±62.56^b^ | 501.21±43.77^b^ | 386.32±41.40 |

ESR, rythrocyte sedimentation rates; CRP, c-reactive protein; WBC, white blood cell counts; GR, Neutrophil counts; LY, Lymphocyte; MO, Monocyte; Crea, serum creatinine; sUA, serum uric acid; ^b^ *P* < 0.05 (in comparison with the Stabilization group).

**Table S3 Real-time fluorescent quantitative PCR primers**.

| **Gene names** | **Forward primer sequence** | **Reverse primer sequence** |
| --- | --- | --- |
| **β-actin** | 5ʹ-GAGCTACGAGCTGCCTGACG -3 | 5ʹ-GTAGTTTCGTGGATGCCACAG -3ʹ |
| **circ_0058051** | 5ʹ- CACCACTTCACGATGCAGCC -3ʹ | 5ʹ-CCTCCTAAACACACAGGCTCTCT-3ʹ |
| **ATG7** | 5ʹ-ATGATCCCTGTAACTTAGCCCA -3ʹ | 5ʹ-CACGGAAGCAAACAACTTCAAC-3ʹ |
| **IL-1β** | 5ʹ-CAACTAGTTGCTGGATACTTGC-3ʹ | 5ʹ-GCTCAGGTCAGTGATGTTAACT-3ʹ |
| **LC3** | 5ʹ-GCTCGTAGTGTCCGCGAT-3ʹ | 5ʹ-AACATGAGCGAGTTGGTCAAG-3ʹ |
